# Draft Genome Sequence of *Chelonobacter oris* Strain 1662^T^, Associated with Respiratory Disease in Hermann’s Tortoises

**DOI:** 10.1128/genomeA.01322-14

**Published:** 2014-12-18

**Authors:** Egle Kudirkiene, Mie Johanne Hansen, Anders Miki Bojesen

**Affiliations:** Department of Veterinary Disease Biology, Faculty of Health and Medical Sciences, University of Copenhagen, Copenhagen, Denmark

## Abstract

*Chelonobacter oris* 1662^T^ is a type strain of the recently described species of the *Pasteurellaceae* family. The strain was isolated from the choanae of a captive tortoise with signs of respiratory tract infection. The genome reported here is approximately 2.6 Mb in size and has a G+C content of 47.1%.

## GENOME ANNOUNCEMENT

*Pasteurellaceae*, with few exceptions, are primarily opportunistic pathogens. Until 2011, the *Pasteurellaceae* family included 15 genera (1); however, new genera isolated from various animal species are continuously added to the family *Pasteurellaceae* (2–6). Most *Pasteurellaceae* species are host-specific and only occasionally may be transmitted between different hosts (7).

*Chelonobacter oris*, currently the only species in the *Chelonobacter* genus, was described in 2009 (8). Based on the phylogeny inferred from analyzing 16S rRNA, *rpoB*, *infB*, and *recN* gene sequences, *C. oris* clusters together with [*Pasteurella*] *testudinis*, a species also found in captive tortoises (9). The two taxa form a distinct cluster distantly related to all other species within the *Pasteurellaceae* family (3).

The genomic DNA from *C. oris* strain 1662^T^ was isolated using a blood and tissue kit (catalog no. 69506; Qiagen). The draft genome sequencing was performed at the National High-Throughput DNA Sequencing Center of the University of Copenhagen. The MiSeq instrument (Illumina) was used for the genome sequencing with a 150-bp paired-end-read format. The CLC Genomic Workbench version 6.5.1 software package (CLC, Denmark) was used to perform quality trimming and *de novo* assembly of the reads. The run yielded 1,156,558 high-quality filtered sequences in pairs containing 134,646,107 bases, which provided an average of 50-fold coverage of the genome. The assembly resulted in 35 contigs ranging from 1,370 bp to 447,435 bp in size. Prediction of protein-coding sequences and annotation was performed by the NCBI Prokaryotic Genomes Automatic Annotation Pipeline (http://www.ncbi.nlm.nih.gov/genomes/static/Pipeline.html). The total estimated size of the genome was 2,610,727 bp with a G+C content of 47.1%. The genome contained 2,284 coding DNA sequences (CDSs), 3 rRNAs, and 48 tRNA sequences. In comparison to other *Pasteurellaceae* members, *C. oris* has one of the largest genomes reported and a relatively high G+C content (10).

All *C. oris* strains are β-hemolytic on calf-blood agar. To identify the potential hemolysins, we compared the assembled genome to a local database built from RTX-like proteins present in different gammaproteobacteria (11), and to the MVirDB database (12) with BLASTp. RTX toxins are the most abundant toxins among *Pasteurellaceae* bacteria, where they often act as the primary virulence factor (7). Although several proteins (locus tags: OA57_07455, OA57_11040, OA57_08905, and OA57_11380) harboring RTX repeats were identified in *C. oris*, none of these showed significant sequence similarity to known RTX toxins. Interestingly, another possible hemolysin (locus tag OA57_07415), together with a channel-forming transporter protein (locus tag OA57_07410) was identified. Both proteins showed significant sequence similarity to the HpmA (accession no. P16466.1) and HpmB (accession no. P16465.1) proteins, respectively, of *Proteus mirabilis* (13). The HpmA protein belongs to the family of the pore-forming calcium-independent cytolysins described previously (14). Homologs of these proteins were not detected in other members of *Pasteurellaceae*, and thus their role in the pathogenicity of *C. oris* remains to be investigated.

### Nucleotide sequence accession numbers.

This whole-genome shotgun project has been deposited at DDBJ/EMBL/GenBank under the accession number JSUM00000000. The version described in this paper is version JSUM01000000.
